# Methodological approach to the *ex vivo* expansion and detection of *T*. *cruzi*-specific T cells from chronic Chagas disease patients

**DOI:** 10.1371/journal.pone.0178380

**Published:** 2017-05-26

**Authors:** Gonzalo R. Acevedo, Silvia A. Longhi, Alcinette Bunying, Nazila Sabri, Augusto Atienza, María P. Zago, Radleigh Santos, Valeria A. Judkowski, Clemencia Pinilla, Karina A. Gómez

**Affiliations:** 1 Instituto de Investigaciones en Ingeniería Genética y Biología Molecular “Héctor N. Torres” (INGEBI), Consejo Nacional de Investigaciones Científicas y Tecnológicas (CONICET), Buenos Aires, Argentina; 2 Torrey Pines Institute for Molecular Studies (TPIMS), San Diego, California, United States of America; 3 Hospital General de Agudos J.M. Ramos Mejia, Buenos Aires, Argentina; 4 Instituto de Patología Experimental (IPE-UNSA), Salta, Argentina; 5 Torrey Pines Institute for Molecular Studies (TPIMS), Port St. Lucie, Florida, United States of America; Institut de recherches cliniques de Montreal, CANADA

## Abstract

The discovery of T cell epitopes is essential not only for gaining knowledge about host response to infectious disease but also for the development of immune-intervention strategies. In Chagas disease, given the size and complexity of the *Trypanosoma cruzi* proteome and its interaction with the host’s immune system, the fine specificity of T cells has not been extensively studied yet, and this is particularly true for the CD4^+^ T cell compartment. The aim of the present work was to optimize a protocol for the generation of parasite-specific memory T cell lines, representative of their *in vivo* precursor populations and capable of responding to parasite antigens after long-term culture. Accordingly, peripheral blood mononuclear cells (PBMC) from both chronic asymptomatic and cardiac patients, and from non-infected individuals, underwent different *in vitro* culture and stimulation conditions. Subsequently, cells were tested for their capacity to respond against *T*. *cruzi* lysate by measuring [^3^H]-thymidine incorporation and interferon-γ and GM-CSF secretion. Results allowed us to adjust initial *T*. *cruzi* lysate incubation time as well as the number of expansions with phytohemagglutinin (PHA) and irradiated allogeneic PBMC prior to specificity evaluation. Moreover, our data demonstrated that parasite specific T cells displayed a clear and strong activation by using *T*. *cruzi* lysate pulsed, Epstein-Barr virus (EBV)-transformed human B lymphocytes (B-LCL), as autologous antigen presenting cells. Under these culture conditions, we generated a clone from an asymptomatic patient’s memory CD4^+^ T cells which responded against epimastigote and trypomastigote protein lysate. Our results describe a culture method for isolating *T*. *cruzi* specific T cell clones from patients with Chagas disease, which enable the acquisition of information on functionality and specificity of individual T cells.

## Introduction

Antigen specific CD4^+^ and CD8^+^ T cells are key components of the immune response developed by chronic patients infected with *Trypanosoma cruzi*, the causative agent of human Chagas disease. Based upon observations in human infection and in experimental models, it is currently accepted that T cells play an important role not only in controlling parasite burden but also modulating disease progression [1–3]. During the chronic phase of the disease, in its asymptomatic or cardiac manifestation forms, CD8^+^ T cells are believed to be involved in both parasite control and in tissue damage, which contributes to cardiac alterations. This dual action could be attributed to the existence of distinct populations of these cells that shift their functional profile during the course of Chagas disease. In fact, asymptomatic patients have a higher frequency of specific interferon-γ (IFN-γ) producing CD8^+^ T lymphocytes than people with severe cardiac symptoms [1,4]. Furthermore, as a consequence of decades of low but continuous antigen exposure, in human *T*. *cruzi* infection, CD8^+^ T cells are driven to exhaustion, leading to progressive impairment of their effector function [1,5–7].

On the other hand, CD4^+^ T cells and monocytes/macrophages participate in the secretion of both inflammatory and anti-inflammatory cytokines, and this release correlates with the clinical outcome of the disease [2,3]. In general, peripheral blood mononuclear cells (PBMC) from cardiac chagasic patients produce more IFN-γ and less IL-10 than do those from asymptomatic patients [8–10]. Accordingly, the majority of recombinant *T*. *cruzi* proteins or total lysate induce a Th1 type cytokine profile (IFN-γ, TNF-α) with suppression of Th2 type cytokines (IL-4, IL-10) in cardiac patients [11–18]. However, we recently demonstrated that this is not true for the immune response developed by *T*. *cruzi* ribosomal P proteins, since the cytokines released upon their stimulation made it difficult to determine a specific Th cell phenotype [18].

Although significant information has been obtained by studying activation markers and cytokines secreted by CD4^+^ and CD8^+^ T cells during *T*. *cruzi* infection [3], knowledge about the fine specificity of these cells is restricted to a few parasite epitopes. Most of these are peptides from proteins belonging to the trans-sialidase family, like TS, ASP-1, ASP-2 [19–21]. Some other examples are comprised within the sequences of cruzipain, MASP, KPM-11, Tc24 or hypothetical proteins TcG1, TcG2 and TcG4 [22–27]. The study of T cell repertoire in protozoan parasitic infections is notably dissimilar from those of viruses and bacteria, in that their large genomes and the complexity of their proteomes hamper the finding of relevant epitopes. In the particular case of *T*. *cruzi*, its haploid genome encodes more than 12,000 genes [28], providing thousands of potential epitopes that could bind to the class I and class II major histocompatibility complex (MHC) molecules and be presented on the surface of infected host cells or antigen presenting cells.

The study of pathogen specific human T cells is difficult due to their low frequency in PBMC samples, approximately 1 in 10^5^ cells in bulk cell population [29]. The potentially large ratio of non-specific to specific cells, together with the protocol used for T cell expansion could result in the selection of T cell populations different from the ones of interest in terms of specificity, phenotype or function. More importantly, the effects of extended long-term *in vitro* culture on T cell phenotype and function may therefore hinder the correlation of many T cell clonal attributes with real *in vivo* characteristics [30].

As part of our ongoing studies to identify parasite epitopes recognized by human T cells in the context of chronic Chagas disease, we decided to optimize the culture conditions of PBMC from asymptomatic and cardiac patients in order to enable a maximum yield of *T*. *cruzi*-specific T cells. In the present work, we analyzed the effect of different time lengths of initial *T*. *cruzi* lysate stimulation, the number of PHA expansions and the usefulness of Epstein-Barr virus (EBV)-transformed human B lymphocytes (B-LCL) as autologous antigen presenting cells, in a small cohort of chagasic patients and non-infected donors. In addition, we generated clones of memory CD4^+^ T cells from an asymptomatic patient that recognize the epimastigote and trypomastigote/amastigote forms of the parasite.

## Materials and methods

### Ethics statement

The research followed the tenets of the Declaration of Helsinki and of the Medical Ethics Committee of the Hospital General de Agudos J.M. Ramos Mejia, which approved the protocols used in this study. All enrolled patients were of age at the time the sample was taken, and they gave written informed consent, according to the Hospital’s Ethics Committee guidelines, before blood collection and after the nature of the study was explained.

### Study population

Patient selection was conducted at the Cardiovascular Division of the Hospital General de Agudos J.M. Ramos Mejia, Buenos Aires, Argentina. The study population consisted of 11 patients with positive serology for Chagas disease, determined by two or more tests (indirect immunofluorescence, ELISA, indirect hemagglutination). The patients, in the chronic phase of the infection, underwent a complete clinical and cardiological examination and were classified as asymptomatic (without demonstrable pathology) or cardiac patients. Five non-*T*. *cruzi* infected individuals with negative serological tests for Chagas disease were included as the control group.

### Parasite lysate

Whole antigenic lysate from *T*. *cruzi* epimastigote was prepared from axenic cultures (CL Brenner strain) in LIT medium, as previously described [18]. After lysis, the suspension was filter sterilized through a 0.2 μm pore-size membrane, aliquoted, and stored at -80°C until use. Trypomastigote/amastigote form lysate was similarly prepared and sterilized, from *T*. *cruzi* Sylvio infected C2C12 cells (MOI 3:1) supernatants. Lysate composition was typically 52–54% trypomastigotes, 46–48% amastigotes (percentages calculated on absolute parasites numbers).

### PBMC isolation

PBMCs were obtained from whole blood by Ficoll-Hypaque density gradient centrifugation (GE Healthcare Bio-Sciences AB, Uppsala, Sweden) according to manufacturer’s instructions, less than 4 h after collection in EDTA-anticoagulated tubes. Cells were resuspended in T cell medium (RPMI-1640 medium, 100 U/ml penicillin, 100 μg/ml streptomycin, 2 mM L-glutamine and 5% heat-inactivated male AB Rh-positive human serum (HS; Sigma, St Louis, MO, USA)) and directly used in experiment, or in fetal calf serum (FCS; Natocor, Córdoba, Argentina) containing 10% dimethylsulfoxide (DMSO), and cryopreserved in liquid nitrogen.

### Generation and antigen priming of Epstein-Barr immortalized B cell lines

Autologous lymphoblastoid B cell lines (B-LCL) were generated for each patient, following an adapted version of a protocol described elsewhere [31]. Briefly, Epstein-Barr virus (EBV) suspension was prepared from B95.8 marmoset lymphoid cells culture supernatant, by centrifugation for 10 min at 300 ×g, 4°C, and filtration through 0.45 μm pore size membrane filter. The resulting virus-containing supernatants were preserved at -80°C until use. For immortalization, 10^7^ PBMC were incubated with 1.25 ml of EBV suspension in a final volume of 5 ml LCL medium (RPMI-1640 supplemented with 100 U/ml penicillin, 100 μg/ml streptomycin, 2 mM L-glutamine and 10% FCS), for 2 h at 37°C. Then, 5 ml of the same medium containing cyclosporine A (Sigma, St Louis, MO, USA) at a final concentration of 1 μg/ml were added, and incubation was continued for 3 weeks. Immortalization success was evaluated during this period by monitoring cultures for cell clumps formation. After this, cells were characterized by flow cytometry, using PE-Cy5-conjugated anti-CD19 and FITC-conjugated anti-CD3 antibodies (BD Biosciences, San Diego, CA, USA.) Immortalized B cell lines (B-LCL) were further expanded in LCL medium without cyclosporine A and cryopreserved in FCS with 10% DMSO, in liquid nitrogen.

For antigen priming, B-LCL were cultured in low serum content LCL medium (RPMI 1640, 2% FCS, 100 U/ml penicillin, 100 μg/ml streptomycin, 2 mM L-glutamine) at a density of 0.5–1.0×10^6^ cells/ml, for 24 h, then washed by centrifugation for 10 min at 400 ×g in 1.5 ml PBS and incubated with 10 μg/ml *T*. *cruzi* lysate, either for 3 h or overnight, before irradiation.

### PBMC stimulation and culture

For initial antigen stimulation, freshly isolated or cryopreserved PBMC from each subject were seeded in 96-well U-bottom plates and cultured in T cell medium with 2.5 μg/ml *T*. *cruzi* lysate (concentration was chosen based on titration experiments [18]). Non-specific stimulation with 5 μg/ml phytohaemagglutinin (PHA, Sigma, St Louis, MO, USA) was used as positive control condition. The presence of clear signs of cellular proliferation (increase in the cell number and clustering) was evaluated as evidence of normal physiological state of the cells at the beginning of the culture process. Similarly, negative control wells were seeded and left unstimulated, exhibiting no signs of cellular proliferation or activation.

Starting at indicated times after antigen stimulation, recombinant human interleukin-2 (IL-2; Peprotech, Mexico DF, Mexico) was added every 3 to 4 days at a final concentration of 50 IU/ml.

### Memory CD4^+^ T cells separation, stimulation and culture

CD4^+^CD45RO^+^ T cells were isolated from PBMC using the immunomagnetic negative selection EasySep Human Memory CD4^+^ T Cell Enrichment Kit and Magnet (StemCell Technologies, Vancouver, Canada), following manufacturer provided indications. Recovery (40.9±14.1%) and purity (93.6±1.2%) of the enriched population was assessed by flow cytometry, staining with PE-Cy7 anti-CD4 (BioLegend, San Diego, CA, USA), FITC anti-CD3, PE-Cy5 anti-CD8, APC anti-CD45RA and PE anti-CD45RO (BD Biosciences, San Diego, CA, USA) antibodies.

For initial stimulation, memory CD4^+^ T cells were seeded in 96-well plates, in T cell medium, at 5×10^3^ cells/well and incubated with 10^4^ autologous irradiated PBMC and 2.5 μg/ml *T*. *cruzi* epimastigote lysate, or at 10^3^ cells/well and incubated with 10^4^ allogeneic irradiated PBMC from 3 non-related, non-infected donors, 1 μg/ml PHA and 50 IU/ml IL-2. This interleukin was also added on every culture since day 3, every 3 to 4 days, at a final concentration of 50 IU/ml. Half the medium (100 μl/well) was refreshed every 10 to 14 days, beginning at day 15.

### PHA expansion

For non-antigen specific expansion, cells were stimulated with 1.0 μg/ml PHA and 50 IU/ml IL-2, in the presence of 2.5×10^4^ irradiated (4,000 rads) allogeneic PBMC/well from 3 non-infected, non-related donors. Control cultures were carried out in parallel by seeding 16–48 wells with irradiated allogeneic cells only.

### *T*. *cruzi* antigen specificity evaluation

Each culture was tested for their response to *T*. *cruzi* proteins in the presence of antigen presenting cells (APC). Briefly, a fraction of the cells were transferred to a new 96-well plate and cultured in T cell medium, with 2.5 μg/ml *T*. *cruzi* lysate or no antigen. As APC, irradiated autologous PBMC (4,000 rads) or irradiated autologous, antigen-primed B-LCL (10,000 rads) were used.

After 18 h, supernatants were collected for the quantification of IFN-γ and GM-CSF production (OptEIA^™^ Human IFN-γ and Human GM-CSF ELISA sets, BD Pharmingen, San Diego, CA, USA). Subtracted medium was replaced with T cell medium and cells were pulsed for 24 h with 0.5 μCi/well [methyl-^3^H]-thymidine (Perkin Elmer, Waltham, MA, USA). Proliferation was measured as incorporated radioactivity, assessed by liquid scintillation counting.

### Limiting dilution assay (LDA)

T cells were plated in 96-well plates, at densities of 10, 3, 1 and 0.3 cells/well, and PHA expansion was carried out, as described above. IL-2 was added every 3 to 4 days at 50 IU/ml. After 2 or 3 subsequent PHA expansions, spaced by 2 to 3 weeks and depending on cell growth, aliquots of these cultures were evaluated for *T*. *cruzi* antigens specificity. Based on this, parasite specific cultures which grew to large enough numbers were characterized for TCR-Vβ expression by flow cytometry (see below).

### TCR-Vβ flow cytometry staining

TCR-Vβ repertoire was analyzed by flow cytometry, using the IOTest Beta Mark Kit (Beckman-Coulter, USA) to stain 5×10^5^ cells/tube, and following manufacturer’s instructions for the staining protocol. CD3 and CD4 expression was also assessed for the same samples, as described for memory CD4^+^ T cells.

## Results

### Impact of different culture conditions on the *in vitro* expansion and functionality of parasite specific T cells

**Fig 1:**
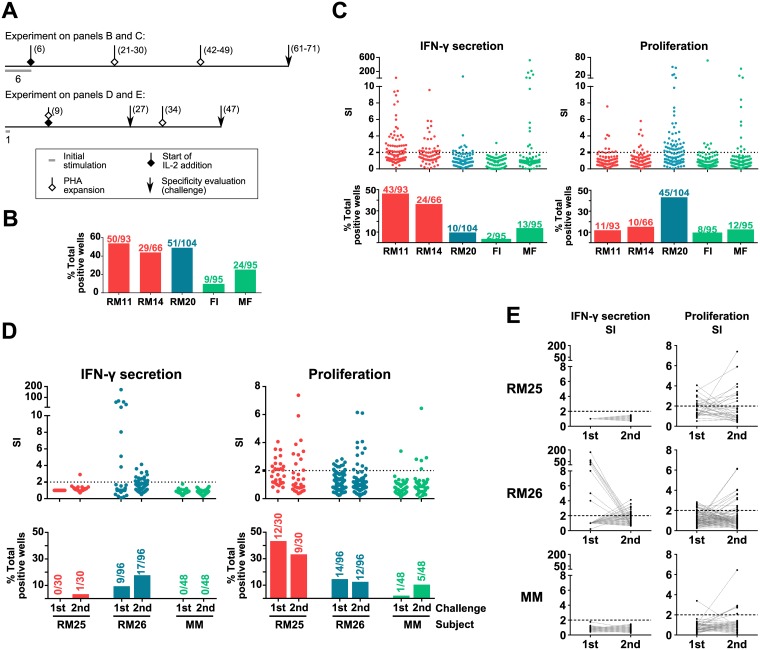
Effect of initial stimulation time and PHA expansions on *in vitro* T cell response against *T*. *cruzi* lysate. PBMC were stimulated and expanded, and aliquots of 1.0–2.0×10^4^ T cells from these cultures were evaluated for *T*. *cruzi* antigens specificity, using 2.0–4.0×10^4^ autologous irradiated PBMC as APC. Response was measured as IFN-γ secretion and proliferation. Stimulatory index (SI) was calculated for each culture well as the response against *T*. *cruzi* lysate divided by the cells baseline response (media only condition). Cultures considered positive were those with an SI≥2 (dotted line). Scatter plots show individual well SI values. Bars represent the percentage of positive wells among the total wells analyzed for each subject. The numbers of positive *vs* total studied wells is indicated above the bars. Color codes indicate which group each subject belongs to: chronic Chagas cardiopathy (red, subjects RM11, RM14 and RM25), asymptomatic Chagas disease (blue, subjects RM20 and RM26) or non-infected (green, subjects FI, MF and MM). **A**. Timeline representation of stimulation and specificity evaluations protocols. Numbers in brackets next to symbols indicate the day (or range of days, when slight variations were required between subjects due to cell growth differences) since protocol start at which each step was performed. Numbers under the ‘initial stimulation’ indicator (grey block) refer to the duration of the stimulus. **B**. Percentage of positive wells for at least one of the readouts in each subject’s cultures when PBMC were initially stimulated for 6 days with *T*. *cruzi* lysate. IL-2 was added every 3–4 days since day 6 post initial stimulation. Specificity evaluation was performed between days 64–71 after initial stimulation, and after 2 PHA expansions. **C**. Wells response by readout for each subject, in the same experiment as B. **D**. *T*. *cruzi*-specific response in cultures derived from two Chagas disease patients and one non-infected subject. Cells were stimulated with *T*. *cruzi* lysate for 1 day, and challenged at day 27 after one PHA expansion (“1^st^ challenge”) or at day 47 after two PHA expansions (“2^nd^ challenge”). IL-2 was added since day 9, and every 3–4 days. **E**. Paired IFN-γ secretion and proliferation parasite-specific responses for each well, on 1^st^ and 2^nd^ challenges, from the same experiment as D. Values are expressed as SI. Statistical analysis for the percentages of positive wells shown in panels B and C are detailed in S1 Table, and for panel D in S2 Table.

With the aim of expanding *T*. *cruzi* specific T cell populations from Chagas disease patients’ PBMC, different culture conditions were tested (Fig 1A).

Overall, the protocol consisted in an initial stimulation with *T*. *cruzi* lysate to selectively amplify the cells of interest and subsequent expansions with PHA, IL-2 and irradiated allogeneic feeder cells to increase cell numbers. To assess the success of this procedure, cells were challenged with *T*. *cruzi* lysate and the response was evaluated by two different readouts, IFN-γ secretion and proliferation. Based on previous findings [18,32], we tested 6 days as initial stimulation time in cultures from cardiac (RM11 and RM14) and asymptomatic (RM20) patients. Two non-infected individuals (FI and MF) were included as controls. As shown in Fig 1B, cultures from infected patients showed 43.9–53.8% positive wells, clearly suggesting the expansion of parasite specific T cells. Under the same conditions, a substantial number of positive wells were detected in cultures from non-infected individuals, in percentages ranging between 9.5–25.3% of the seeded wells (Fig 1B and 1C). However, the values obtained for the infected subjects were significantly higher (S1 Table).

Given that the response detected in cultures from non-infected subjects could be associated with undesired *in vitro* activation and amplification of naïve T cells, a following experiment was carried out using a 1-day initial stimulation. This modification led to a reduction in the percentage of positive wells from a non-infected donor’s cells (MM), but affecting the reactivity of cultures from asymptomatic patient RM26 and cardiac patient RM25 as well (Fig 1D). Overall, these results suggest that a shorter initial *T*. *cruzi* lysate stimulation period (1 day) leads to a lower *in vitro* activation of cell populations responsive to parasite antigens, which could involve naïve, *in vitro* activated T cells, but also memory, specific T cells.

Aiming to address how PHA expansion cycles impacts on *T*. *cruzi* specific T cells enrichment and their functional profile and capability, antigen specificity evaluations were performed after 27 days of culture and 1 PHA expansion (“1^st^ challenge”), or after 47 days of culture and 2 PHA expansions (“2^nd^ challenge”, Fig 1D and 1E). In terms of IFN-γ secretion, specific response was observed only for the asymptomatic chronic Chagas patient RM26. For this subject, as shown in Fig 1D, the percentage of positive wells increased after two rounds of PHA expansion, but the overall SI levels were lower, as compared with the values obtained after a single PHA expansion. On the other hand, a smaller percentage of proliferation positive wells, but higher SI values, were observed for both chronic Chagas patients after two PHA expansions. However, for the non-infected subject, positive wells rose from 2.0% after one round, to 10.1% after two rounds of PHA expansion, suggesting that several non-antigen specific stimulations might favor the undesired amplification of T cells that are indeed not related to infection.

Interestingly, many of the wells that responded on the 1^st^ challenge became negative on the 2^nd^ and vice versa, for both proliferation and IFN-γ secretion, as plotted on Fig 1E. No wells switched from IFN-γ positive to proliferation positive, while 5.0% of the RM26 wells switched from proliferation positive to IFN-γ positive. Of these, only 1.0% (on total seeded wells) remained responsive by IFN-γ secretion while gaining proliferative responsiveness (data not shown). Wells from neither the cardiac patient nor the non-infected individual exhibited this behavior.

Overall, these results show that changing the conditions of the initial *T*. *cruzi* stimulation and subsequent PHA expansion has an evident impact on the *in vitro* amplification of *T*. *cruzi* specific cells. Furthermore, the data obtained with non-infected individuals indicate that there is a significant and sizeable population of either naïve T cells, or memory T cells with a different specificity, but cross-reactive to *T*. *cruzi* antigens, that can be expanded *in vitro* under these culture conditions.

### Autologous immortalized B-cells as antigen presenting cells of *T*. *cruzi* lysate to T cell cultures

**Fig 2:**
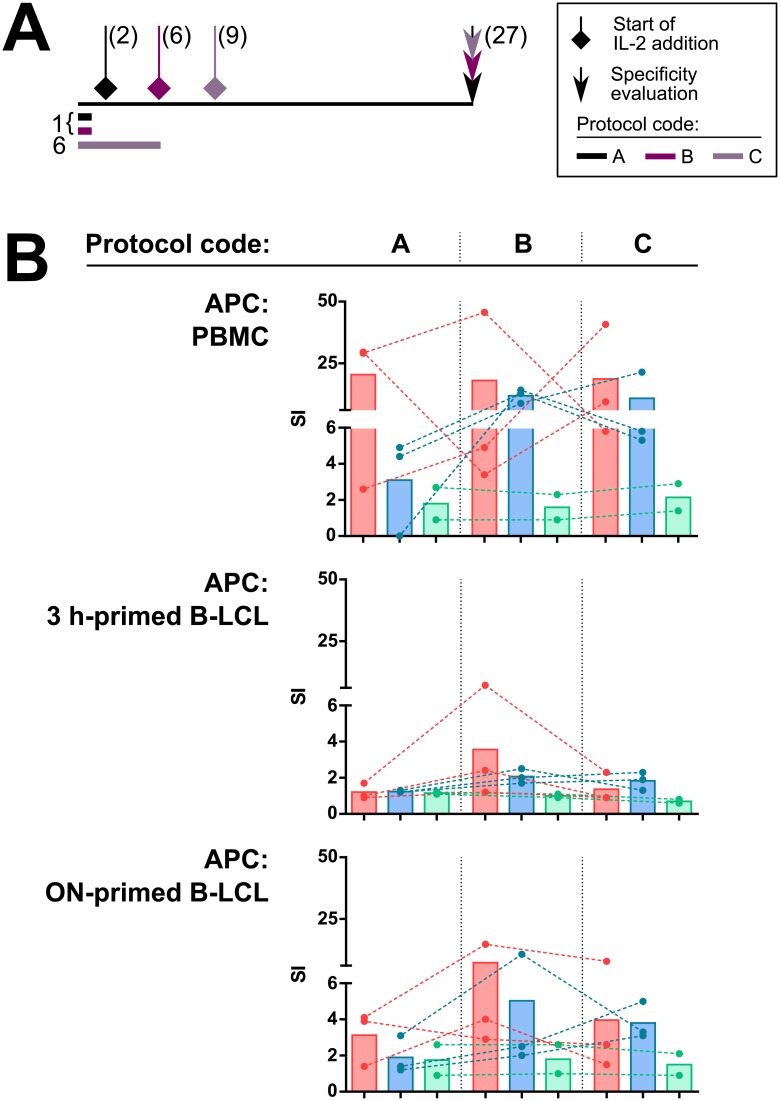
Use of autologous Epstein-Barr immortalized B-cells as antigen presenting cells. PBMC from three cardiac patients, three asymptomatic patients and two non-infected individuals were seeded in two wells of 96-well plates at 2×10^5^ cells/well. Three different protocols (namely A, B and C) were used. IL-2 was added every 3–4 days after the indicated time. At day 27 post-stimulation, cells from each condition were pooled and 2×10^3^ T cells from each of these cultures were challenged with *T*. *cruzi* lysate or culture media only, using autologous PBMC (4×10^3^ cells/well), 3 h-antigen primed autologous B-LCL, or overnight-antigen primed autologous B-LCL as APC (4×10^3^ cells/well). **A**. Timeline representation of protocols A, B and C. Numbers in brackets next to symbols indicate the day since protocol start at which each step was performed. Numbers under the ‘initial stimulation’ indicator (grey blocks) refer to the duration of the stimulus. **B**. IFN-γ secretion response as measured by ELISA. For each subject, stimulatory index (SI) was calculated as the response against *T*. *cruzi* lysate divided by the cells baseline response (media only condition), each dot represents mean SI value of three biological replicates for a single subject. Dashed lines link values belonging to the same subjects. Bars represent mean SI value for each group. Color codes indicate which group each subject belongs to: chronic Chagas cardiopathy (red), asymptomatic Chagas disease (blue) or non-infected (green).

Due to the limited availability of patients’ PBMC, another important factor to consider when developing T cell lines and clones for using in antigen discovery research is which cells are to be used as antigen presenting cells. In order to assess the capability of autologous Epstein-Barr virus-immortalized B cells (B-LCL) to present *T*. *cruzi* antigens in specificity evaluation experiments, T cell culture protocols were carried out (Fig 2A), emulating stimulation and culture conditions from experiments in Fig 1. To load the B-LCL, two different antigen priming conditions were tested (3 h and overnight) by pre-incubating these cells with the lysate at a concentration of 10 μg/ml, prior to the challenge experiment.

Although the response was evaluated by IFN-γ secretion and proliferation, only the former type of response was detectable upon challenge. As shown in Fig 2B, although pre-incubation of the B-LCL with *T*. *cruzi* lysate induced IFN-γ secretion in T cell cultures from most subjects studied, none of the cultures challenged with lysate primed-B-LCL reached the level of activation induced by irradiated PBMC as APC. However, the overnight priming condition produced SI values that enable a better detection of the response against the parasite lysate in chronic Chagas patient cultures, as compared to the 3 h priming condition. Additionally, the quality of the response was higher in protocols B (1 day of antigen stimulation with IL-2 added since day 9) and C (6 days of antigen stimulation with IL-2 added since day 6) when B-LCL were used as APC. In conclusion, the results presented here demonstrate that overnight primed B-LCL can be used as APC to present *T*. *cruzi* antigens to T cells.

### Generation of *T*. *cruzi* specific memory CD4^+^ T cell lines from an asymptomatic patient

**Fig 3:**
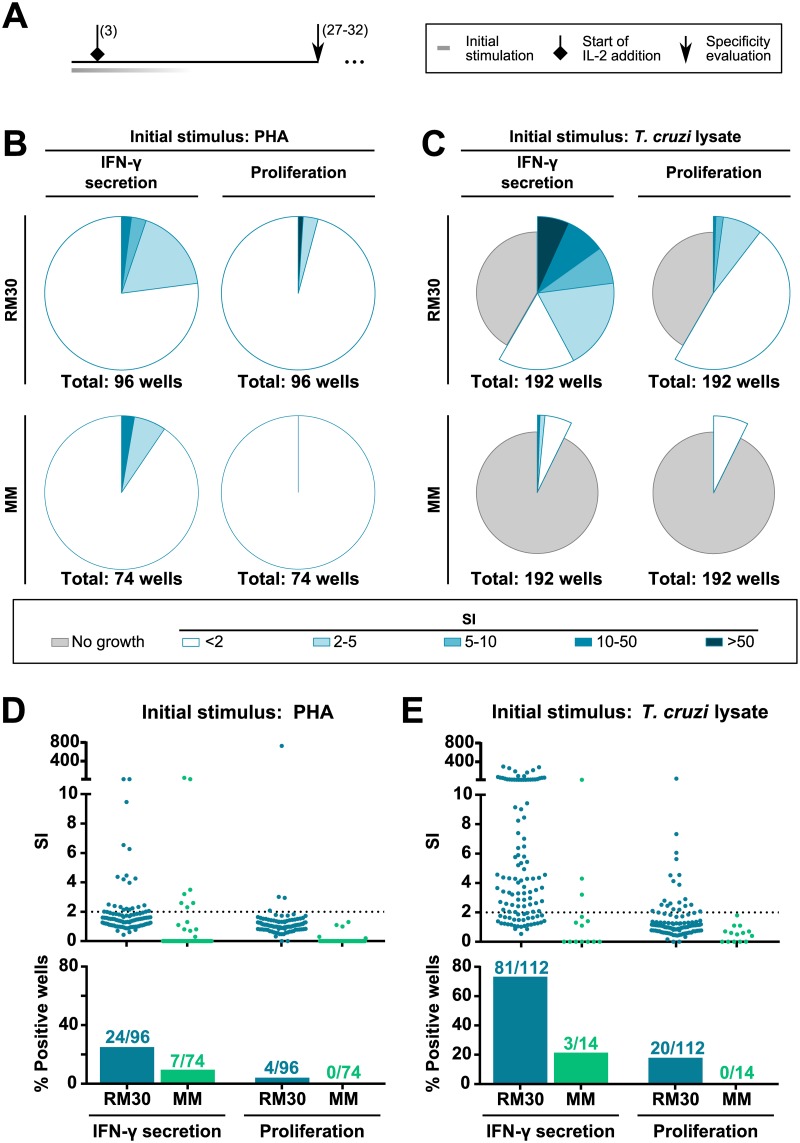
Effect of stimulus used for initial expansion of antigen specific memory CD4^+^ T cells and their *in vitro* response against *T*. *cruzi* antigens. Sorted memory CD4^+^ T cells from an asymptomatic Chagas patient (RM30) and a non-infected subject (MM) were stimulated with PHA (B, D) or parasite lysate (C, E) as detailed under Materials and Methods. Five thousand cells from each culture well were challenged between days 27–32 (depending on cell growth) with parasite lysate or culture media only and the antigen-specific response was measured as IFN-γ secretion and proliferation. Ten thousand autologous overnight-primed B-LCL per well were used as APC. SI was calculated for each well as the response against *T*. *cruzi* lysate divided by the cells baseline response (media only condition). Statistical analysis of the percentage of positive wells is shown in S3 Table. **A**. Timeline representation of stimulation and challenge protocols. Numbers in brackets next to symbols indicate the day (or range of days) since protocol start at which each step was performed. **B, C**. Pie chart representations of the wells that showed cell growth after initial stimulation, and different degrees of antigen specific response against *T*. *cruzi* lysate. **D, E**. SI values (scatter plots) and percentage of positive wells (bars) for each readout. Cultures considered positive were those with an SI≥2 (dotted line). Positive and total studied wells are indicated in numbers above the bars.

Given the results presented above, we hypothesized that using enriched memory T cells, instead of bulk PBMC, would favor the amplification of T cells that emerged *in vivo*, in response to infection. Seeking to contribute to the scarce existing knowledge on the specificity of memory CD4^+^ T cells in Chagas disease, we decided to focus our efforts on this population. For this, CD3^+^CD4^+^CD45RO^+^ cells from an asymptomatic chronic Chagas patient (RM30) and a non-infected individual (MM) were magnetically sorted from PBMC samples, and initially cultured with two different stimuli: *T*. *cruzi* lysate with irradiated autologous PBMC as APC (antigen specific, selective expansion) or PHA, IL-2 and irradiated allogeneic PBMC as feeder cells (non-antigen specific, non-selective expansion). An overview of the protocols used is represented in Fig 3A. The stimulation of cells from both subjects with PHA resulted in a clear growth of all the seeded wells (Fig 3B). In contrast, the percentages of wells that grew in response to *T*. *cruzi* lysate was lower and significantly different between subjects (58.3% for RM30 *vs* 12.4% for MM, Fisher’s exact test, *p*<0.001, Fig 3C).

After the initial expansion, specificity was assessed for these cultures using overnight antigen primed autologous B-LCL as APC. Most of RM30 wells initially stimulated with parasite lysate responded against *T*. *cruzi* antigens (73.2% for IFN-γ secretion, 17.9% for proliferation, Fig 3C and 3E) in contrast to the remarkably lower number found in wells initially expanded with PHA (25.0% for IFN-γ, 4.2% for proliferation, Fig 3B and 3D). On the other hand, 21.0% of the lysate expanded wells (only 1.53% out of the total seeded wells) from the non-infected subject responded by IFN-γ secretion, and none responded by proliferation (Fig 3B and 3D). Fisher’s exact test was applied to analyze the difference in frequencies of wells responding, by at least one of the readouts, between the infected and the non-infected donors, and a statistical significance was observed under both initial stimulation conditions, *T*. *cruzi* lysate (*p* = 0.0003) and PHA (*p* = 0.02). Furthermore, the IFN-γ secretion levels measured in cultures initially expanded with *T*. *cruzi* lysate were significantly higher for the chronic Chagas patient as compared to those from the non-infected individual’s cultures (*p*<0.01, Mann-Whitney test).

**Fig 4:**
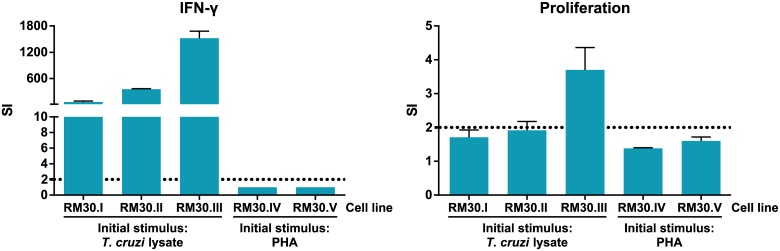
Antigen specific response in selected cultures from subject RM30. Cell lines were established from *T*. *cruzi* specific cultures, based on results from experiment depicted on Fig 3. Cells were submitted to 2 PHA expansion cycles prior to specificity evaluation. Five thousand cells from each culture well were challenged with *T*. *cruzi* lysate, using 10^4^ autologous overnight-primed B-LCL per well as APC. SI was calculated for each well as the response against *T*. *cruzi* lysate divided by the cells baseline response (media only condition), cultures considered positive were those with an SI≥2 (dotted line). Bars show the mean values and standard deviation for three replicates of each measure.

Several T cell lines were generated from cultures detected as parasite-specific, and they were further expanded and re-tested for specificity, as previously described. As shown in Fig 4, lines originated from cultures initially stimulated with *T*. *cruzi* maintained their responsiveness against parasite lysate by IFN-γ secretion, while those expanded from cells stimulated with PHA did not. Moreover, response by proliferation was no longer observed at this point, even from T cell lines RM30.I and RM30.II, which had been positive for this readout on previous specificity evaluations. In order to avoid limiting the evaluation of specificity only to the quantification of IFN-γ, a multiplex cytokine detection assay (MesoScale Discovery, San Diego, CA) was performed on selected supernatants from experiment shown in Fig 3. A panel of 8 inflammatory and regulatory cytokines (IL-2, IL-4, IL-10, IL-13, IL-17a, TNF-α, GM-CSF, IFN-γ) was evaluated. Results from these experiments pinpointed IFN-γ and GM-CSF as the most reliable readouts for *T*. *cruzi* CD4^+^ T cell specificity (data not shown).

In summary, these results confirm the advantages of an initial stimulation with the parasite lysate on memory CD4^+^ T cells obtained from chronic Chagas patients, both in terms of expansion capability and selectivity.

### Generation of *T*. *cruzi* specific memory CD4^+^ T cell clones

**Fig 5:**
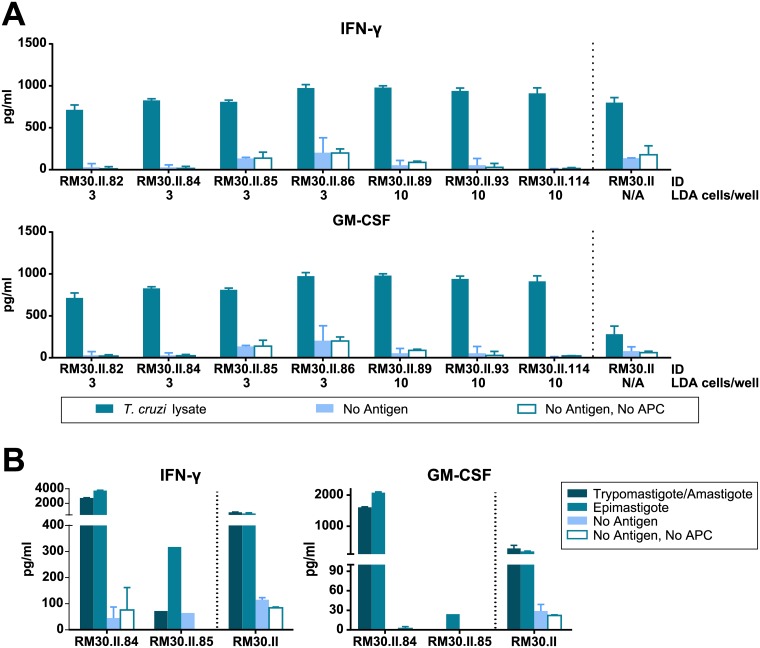
Antigen specific response in cultures resulting from limiting dilution assay (LDA) of culture RM30.II. **A**. Twenty-five thousand cells from each culture well were challenged with *T*. *cruzi* lysate, using 5×10^4^ autologous overnight-primed B-LCL per well as APC. Specificity against *T*. *cruzi* antigens was assessed for several potentially monoclonal lines by IFN-γ and GM-CSF secretion. N/A: Non applicable. **B**. Specific response was tested using lysates from different stages in the parasite’s life cycle (epimastigote and trypomastigote/amastigote) for T cell lines RM30.II.84 and .85.

In order to isolate an antigen-specific clonal population from the T cell lines generated as described above, we used line RM30.II from the previous section to set up a limiting dilution assay (LDA) using 4 different cell density conditions (10, 3, 1 and 0.3 cells/well), in 10 U-bottom 96-well plates for each condition. Of note, RM30.II was selected for this process based on its growth rate, besides the observed specific response. Cells were expanded with PHA 7 times (except for the 10 cells/well condition, which were expanded 5 times), and specificity evaluations were performed, measuring IFN-γ and GM-CSF secretion, as detailed on S1 Fig. Using growth rate and specificity as criteria, several *T*. *cruzi*-specific cultures were selected. On day 99 after the LDA start, all of these cultures showed a clear parasite-specific response (Fig 5A).

In addition, T cell lines RM30.II.84 and .85 were tested for specificity against epimastigote and trypomastigote/amastigote lysate. As shown in Fig 5B, line RM30.II.84 responded specifically to both parasite forms. Seeking to assess whether this line was monoclonal, we performed a TCR-Vβ repertoire characterization by flow cytometry. Results showed these cells exclusively express a TCR from the Vβ 5.2 family, demonstrating RM30.II.84 is a clonal T cell line (Table 1).

**Table 1:** Detected frequencies of TCR-Vβ families expressed on T cells from lines RM30.II and RM30.II.84.

TCR-Vβ family	RM30.II	RM30.II.84
**1**	0.00	0.02
**2**	**0.16**	0.02
**3**	0.00	0.00
**4**	0.00	0.01
**5.1**	**0.39**	0.03
**5.2**	**0.28**	**0.92**
**5.3**	0.02	0.00
**7.1**	0.00	0.02
**7.2**	0.00	0.02
**8**	0.00	0.00
**9**	0.00	0.00
**11**	0.06	0.01
**12**	0.00	0.00
**13.1**	0.03	0.04
**13.2**	0.00	0.00
**13.6**	0.00	0.01
**14**	0.00	0.00
**16**	0.00	0.00
**17**	0.00	0.01
**18**	0.00	0.01
**20**	0.04	0.00
**21.3**	0.01	0.02
**22**	0.00	0.02
**23**	0.00	0.00

## Discussion

It is known that the *in vitro* expansion of lymphocytes collected from peripheral blood mononuclear cells (PBMC) could modify the *ex vivo* phenotype and function of CD4^+^ and CD8^+^ T cells, which in part, could be reflected in the loss of antigen specific memory subsets, changes in specificity and the acquisition of late effector or exhausted phenotypes over time [33–36].

Here, we generated enriched *T*. *cruzi* specific T cell lines after an initial PBMC stimulation with parasite lysate. The frequency of *T*. *cruzi* responsive cell cultures was greater in asymptomatic and cardiac patients than in non-infected individuals, and it increased in a stimulation time dependent manner. Noticeably, when statistical analysis was applied population-wise to each subject’s cultures, only T cells from Chagas disease patients showed a significantly higher response against *T*. *cruzi* lysate, as compared to the baseline response. Conversely, non-infected individuals exhibited either no response, or response inhibition induced by the parasitic antigens (S2 and S3 Figs). However, when bulk PBMC were used to generate the libraries, some of the T cell cultures expanded from non-infected donors’ samples were responsive to *T*. *cruzi*. This response, provoked by whole parasite lysate, could be the result of: 1) lectins or superantigens, which could induce a polyclonal, non-specific activation; 2) an antigen-specific primary response from naïve T cells, or 3) memory T cells being specific for host or other pathogens epitopes, and cross-reactive to *T*. *cruzi* antigens. In this regard, Piuvezam *et al* (1993) demonstrated that short term T cell lines generated against *T*. *cruzi* from healthy donors PBMC were predominantly memory cells and proliferative response was MHC dependent, suggesting that parasite lysate contains epitopes that cross-react with other antigens of environmental origin, and ruling out a potential involvement of lectins or superantigens as non-specific mitogens [37]. Seeking to minimize the potential contribution of naïve *T*. *cruzi* reactive T cells expansion, we undertook our subsequent experiments with enriched memory T cells instead of whole PBMC. Our observation of a certain degree of reactivity in memory CD4^+^ T cells from a non-infected subject favors the hypothesis of cross-reactivity, meaning these cells have most probably been selected *in vivo* at the setup of an adaptive response to non-*T*. *cruzi* related agents, and cross-react against *T*. *cruzi* epitopes. It is also likely that they would be activated early in the context of a first exposure of this subject to the parasite, which makes their specificity an interesting subject for further studies. Nevertheless, under our experimental conditions, the frequency and reactivity of such parasite-responsive cultures were remarkably lower than in those derived from a Chagas disease patient, reinforcing the reliability of our approach.

Another important conclusion drawn from our results lies on the fact that, for memory CD4^+^ T cell cultures, initially stimulated with *T*. *cruzi* lysate, the proportion of wells exhibiting cellular growth was dramatically higher in the chagasic patient RM30 as compared to the non-infected individual MM. On the contrary, when PHA was the initial stimulus, cell growth was observed across all the culture wells, from both subjects. Furthermore, initial stimulation of the Chagas disease patient’s cells with parasite lysate rendered not only a greater proportion of parasite-reactive wells, but also higher specific response values. On that regard, Geiger et al. (2009) demonstrated that within the memory compartment, polyclonal PHA stimulation of CD4^+^ T cells allows the expansion of T cells specific for recall antigens from tetanus, CMV and *M*. *tuberculosis*, at high frequencies [38]. However, even though we were able to reproduce this phenomenon with cells from Chagas disease patients, our results highlight the role of a single initial *in vitro* stimulation with parasite proteins to increase the frequency at which specific cultures were detected, enabling the subsequent expansion and characterization of pathogen-specific T cells. Several other reported protocols using a specific antigen or its peptides as initial stimulus support our finding [39, 40].

To yield an appropriate quantity of specific T cells, another important step is the expansion of the cultures. Our results showed that, when starting with bulk PBMC, two rounds of non-specific re-stimulation with PHA could increase the number of naïve/cross-reactive T cells not related to infection, as shown by the proliferation response rendered by T cells derived from non-infected individuals. In addition, each round affected the specificity of the cell populations. This dynamic change in antigen specificity could also be associated with a direct effect of the mitogen over the functional characteristics of T cells [41], or be the consequence of the enrichment of certain T cells in the well, leading to gain or loss in frequency of parasite specific T cells. The same phenomenon was observed in T cell lines RM30.IV and .V, which came from a chronic asymptomatic Chagas patient enriched memory CD4^+^ T cells with a non-specific initial stimulus. However, the specificity of lines RM30.I, .II and .III, which were generated by antigen-specific stimulation of the same T cell subset seems to be long-term stable, regardless of the number of subsequent PHA expansions.

An interesting finding of the present study lies in the use of *T*. *cruzi* primed B-LCL to function as antigen-presenting cells (APCs) for the assessment of T cell responses. Although autologous PBMC or dendritic cells are a better source of the so called “professional” APC, their usage relies on repeated blood donations [42]. In contrast, although B-LCL have less capacity to present soluble antigens compared to monocytes or dendritic cells, they are a sustainable and scalable source of APC. In fact, immortalized B-LCL have been broadly used with success in different protocols that require *in vitro* antigen presentation to T cells. However, no data were available about their use as APC for the presentation of *T*. *cruzi* proteins. Here, our results showed that both PBMC and overnight parasite lysate-pulsed B-LCL induced similar IFN-γ secretion profiles in T cells from asymptomatic and cardiac patients.

After LDA, T cell lines generated from memory CD4^+^ T cells from a chagasic patient and maintained under the described culture conditions, retained their specific response capability towards *T*. *cruzi* lysate for more than 98 days. Moreover, T cell line RM30.II.84 responded against epimastigote and trypomastigote/amastigote lysate with similar strength, indicating that these cells recognize a common epitope expressed in both non-infective and infective parasite forms. Finally, TCR-Vβ analysis demonstrated a prevalence of TCR-Vβ 2, 5.1 and 5.2 families in memory CD4^+^ T cell line RM30.II while its derived clone RM30.II.84 expresses TCR-Vβ 5.2.

Even though the generality of our findings is limited due to the small number of subjects (patients and non-infected donors) included throughout the study, it is possible to ascertain a suitable protocol for the generation of specific oligo- and monoclonal T cell lines from patients with Chagas disease. Although our first efforts were focused on the generation of memory CD4^+^ T cell clones from an asymptomatic subject, we expect that this method will be useful for the expansion of *T*. *cruzi* specific T cells from cardiac patients. However, it could represent a greater challenge, due to the higher frequency of CD4^+^ T cells with senescent and exhausted phenotype in the peripheral blood of these patients as compared to asymptomatic ones [43]. We speculate that it will not be possible to obtain as many detectable specific T cell lines per patient as we have for asymptomatic patients assayed so far, or that it will be more difficult to amplify them to numbers large enough. Hoping to shed some light in this issue, we are currently looking into the *ex vivo* expression profiles of several inhibitory receptors which have been associated to T cell exhaustion [44], and how they vary during *in vitro* culture and expansion.

In conclusion, our data demonstrate that: 1) starting the expansion from enriched memory T cells instead of PBMC allow the increase of the stimulation time, enabling the selection of a larger number of parasite specific T cell cultures; 2) an antigen specific initial stimulation is beneficial for the *ex vivo* selective expansion of pathogen related memory CD4^+^ T cell populations, as compared with a non-antigen specific, although stronger, stimulation (as PHA); 3) the use of different readouts, in this particular case IFN-γ and GM-CSF secretion, is important in order to detect the largest number of antigen-specific positive wells and 4) B-LCL primed with *T*. *cruzi* lysate by an overnight incubation are capable to perform antigen presentation and stimulate parasite specific T cells. This work constitutes the first step in our ongoing T cell driven approach to the identification of human immunogenic *T*. *cruzi* epitopes, a matter of key relevance for the understanding of the immune response in Chagas disease.

## Supporting information

S1 Fig Schematic representation of the LDA protocol. T cells were plated in 96-well plates, at densities of 10, 3, 1 and 0.3 cells/well (rows), and submitted to PHA expansion. Numbers indicate the amount of wells that were expanded each time (columns), after the previous round of selection, according to the criteria indicated by arrows.
(EPS)

S2 Fig IFN-γ secretion raw values for specificity experiments on T cells derived from PBMC. For each challenge experiment on Fig 1, paired results for each culture were statistically analyzed using Wilcoxon’s signed rank test. (*/#: *p*<0.05; **/##: *p*<0.01; ***/###: *p*<0.001; ****/####: *p*<0.0001). Asterisks show statistical significance in cases were the *T*. *cruzi* lysate challenged response was significantly higher than the one from the culture medium only condition (W>0). Similarly, number signs show significance in the cases in which the baseline response was significantly higher than the one from the lysate challenged aliquots (W<0). Color codes indicate which group each subject belongs to: chronic Chagas cardiopathy (red), asymptomatic Chagas disease (blue) or non-infected (green). NA: No antigen condition; Tc: *T*. *cruzi* lysate. **A**. Results from experiment explained in Fig 1B and 1C. **B**. Results from experiment explained in Fig 1D.
(PDF)

S3 Fig Proliferation raw values for specificity experiments on T cells derived from PBMC. For each challenge experiment on Fig 1, paired results for each culture were statistically analyzed using Wilcoxon’s signed rank test. (*/#: *p*<0.05; **/##: *p*<0.01; ***/###: *p*<0.001; ****/####: *p*<0.0001) Asterisks show statistical significance in cases were the *T*. *cruzi* lysate challenged response was significantly higher than the one from the culture medium only condition (W>0). Similarly, number signs show significance in the cases in which the baseline response was significantly higher than the one from the lysate challenged aliquots (W<0). Color codes indicate which group each subject belongs to: chronic Chagas cardiopathy (red), asymptomatic Chagas disease (blue) or non-infected (green). NA: No antigen condition; Tc: *T*. *cruzi* lysate. **A**. Results from experiment explained in Fig 1B and 1C. **B**. Results from experiment explained in Fig 1D.
(PDF)

S1 Table Statistical analysis for the effect of 6 days stimulation with parasite lysate on PBMC. The numbers correspond to Fisher's exact tests *p* values with Bonferroni-Holm correction for multiple comparisons applied to the analysis of the percentage of positive wells. Data from two non-infected subjects (FI and MF) was pooled for comparison with each infected subject, see Fig 1B and 1C. *p*<0.05 was considered statistically significant.
(DOCX)

S2 Table Statistical analysis for the effect of PHA expansions on *T*. *cruzi* specific T cell response. The numbers correspond to *p* values of Fisher's exact tests with Bonferroni-Holm correction applied to the percentage of positive wells from each patient in comparison with non-infected subject, named MM, see Fig 1D and 1E. *p*<0.05 was considered significantly.
(DOCX)

S3 Table Statistical analysis for the effect of initial stimulus on memory CD4^+^ T cells. The numbers correspond to *p* values of Fisher's exact tests with Bonferroni-Holm correction applied to the percentage of positive wells from the patient in comparison with non-infected subject, named MM, see Fig 3. *p*<0.05 was considered significantly.
(DOCX)
