# Correction: Methodological approach to the *ex vivo* expansion and detection of *T*. *cruzi*-specific T cells from chronic Chagas disease patients

**DOI:** 10.1371/journal.pone.0184467

**Published:** 2017-08-31

**Authors:** Gonzalo R. Acevedo, Silvia A. Longhi, Alcinette Bunying, Nazila Sabri, Augusto Atienza, María P. Zago, Radleigh Santos, Valeria A. Judkowski, Clemencia Pinilla, Karina A. Gómez

The affiliation for the tenth author is incorrect. Karina A. Gómez is not affiliated with #2 but with #1, the Instituto de Investigaciones en Ingeniería Genética y Biología Molecular “Héctor N. Torres” (INGEBI).
