# Supplementary figures and images for: Methodological approach to the ex vivo expansion and detection of T. cruzi-specific T cells from chronic Chagas disease patients
Source: PLoS One. 2017 May 26;12(5):e0178380. doi: 10.1371/journal.pone.0178380 (PMC5446171; doi:10.1371/journal.pone.0178380)

**A**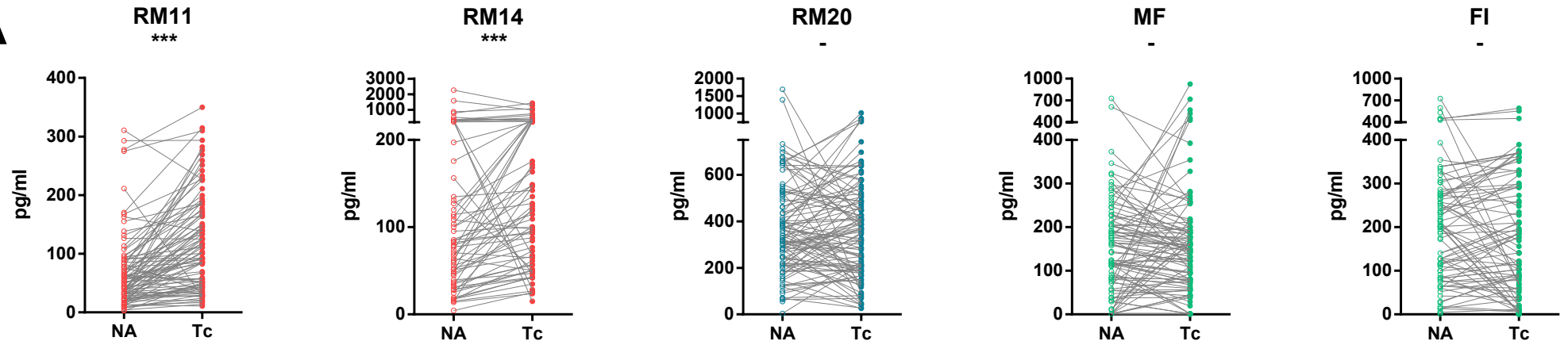**B**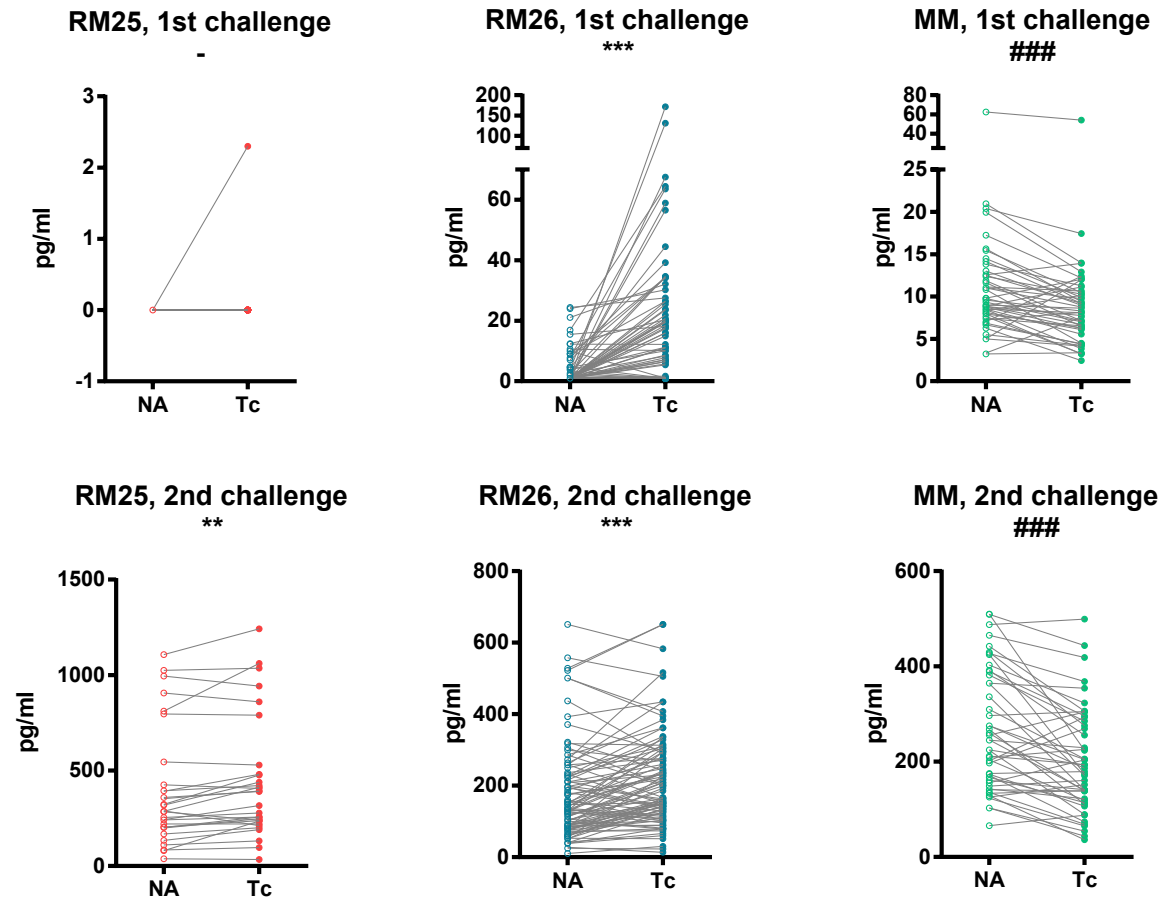

Supplement: S2 Fig — For each challenge experiment on Fig 1, paired results for each culture were statistically analyzed using Wilcoxon’s signed rank test. (*/#: p<0.05; **/##: p<0.01; ***/###: p<0.001; ****/####: p<0.0001). Asterisks show statistical significance in cases were the T. cruzi lysate challenged response was significantly higher than the one from the culture medium only condition (W>0). Similarly, number signs show significance in the cases in which the baseline response was significantly higher than the one from the lysate challenged aliquots (W<0). Color codes indicate which group each subject belongs to: chronic Chagas cardiopathy (red), asymptomatic Chagas disease (blue) or non-infected (green). NA: No antigen condition; Tc: T. cruzi lysate. A. Results from experiment explained in Fig 1B and 1C. B. Results from experiment explained in Fig 1D. (PDF) [file pone.0178380.s002.pdf]

**A**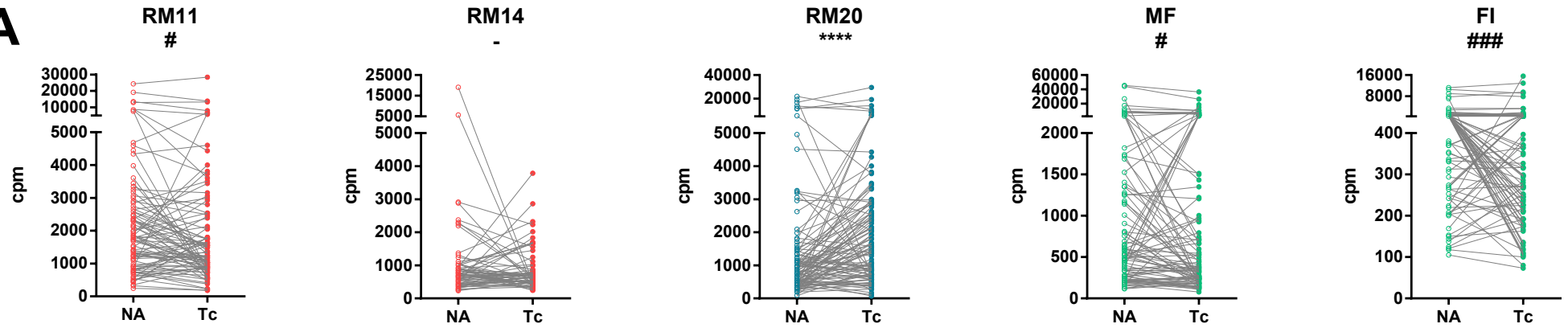**B**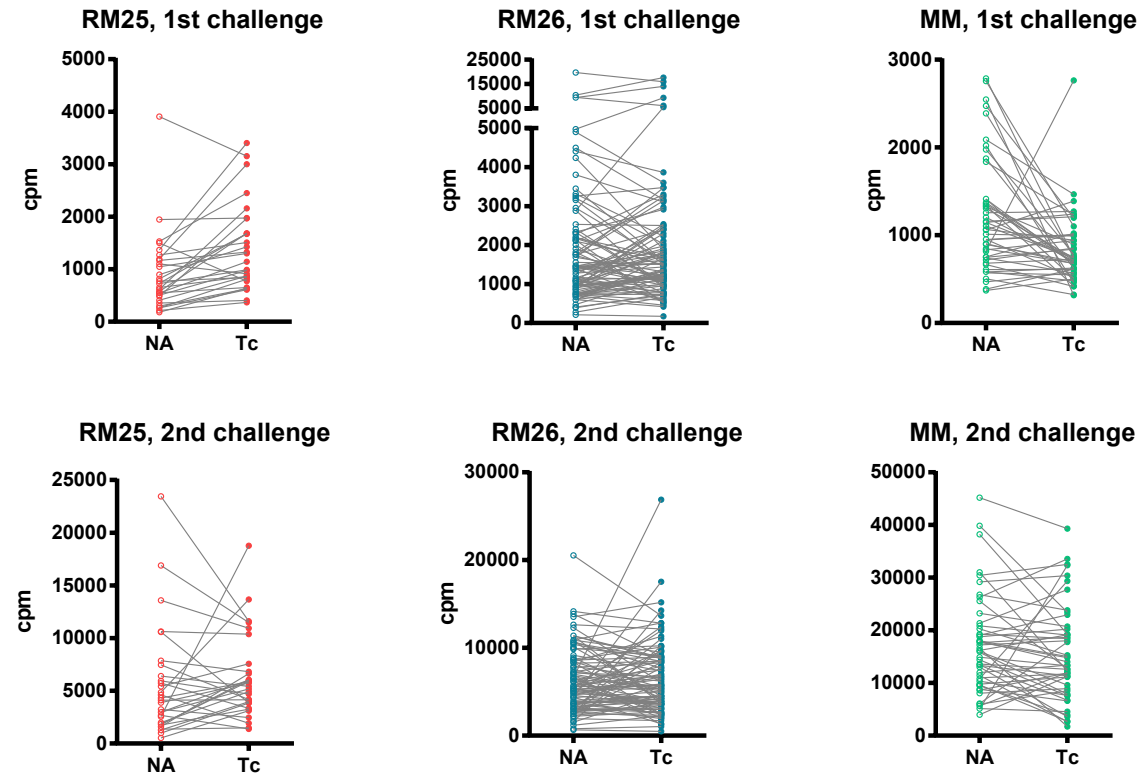

Supplement: S3 Fig — For each challenge experiment on Fig 1, paired results for each culture were statistically analyzed using Wilcoxon’s signed rank test. (*/#: p<0.05; **/##: p<0.01; ***/###: p<0.001; ****/####: p<0.0001) Asterisks show statistical significance in cases were the T. cruzi lysate challenged response was significantly higher than the one from the culture medium only condition (W>0). Similarly, number signs show significance in the cases in which the baseline response was significantly higher than the one from the lysate challenged aliquots (W<0). Color codes indicate which group each subject belongs to: chronic Chagas cardiopathy (red), asymptomatic Chagas disease (blue) or non-infected (green). NA: No antigen condition; Tc: T. cruzi lysate. A. Results from experiment explained in Fig 1B and 1C. B. Results from experiment explained in Fig 1D. (PDF) [file pone.0178380.s003.pdf]
