# Supplementary material for: Methodological approach to the ex vivo expansion and detection of T. cruzi-specific T cells from chronic Chagas disease patients
Source: PLoS One. 2017 May 26;12(5):e0178380. doi: 10.1371/journal.pone.0178380 (PMC5446171; doi:10.1371/journal.pone.0178380)
Supplement: S1 Table — The numbers correspond to Fisher's exact tests p values with Bonferroni-Holm correction for multiple comparisons applied to the analysis of the percentage of positive wells. Data from two non-infected subjects (FI and MF) was pooled for comparison with each infected subject, see Fig 1B and 1C. p<0.05 was considered statistically significant. (DOCX) [file pone.0178380.s004.docx]

**S1 Table:**

**Statistical analysis for the effect of 6 days stimulation with parasite lysate on PBMC**

| **Patient** | **IFN-γ** | **Proliferation** | **At least one readout** |
| --- | --- | --- | --- |
| RM11 | **<0.0001** | 0.443 | **<0.0001** |
| RM14 | **<0.0001** | 0.427 | **<0.0001** |
| RM20 | 0.381 | **<0.0001** | **<0.0001** |

The numbers correspond to Fisher's exact tests *p* values with Bonferroni-Holm correction for multiple comparisons applied to the analysis of the percentage of positive wells. Data from two non-infected subjects (FI and MF) was pooled for comparison with each infected subject, see Fig 1 B and C. *p*<0.05 was considered statistically significant.
