# Supplementary material for: Methodological approach to the ex vivo expansion and detection of T. cruzi-specific T cells from chronic Chagas disease patients
Source: PLoS One. 2017 May 26;12(5):e0178380. doi: 10.1371/journal.pone.0178380 (PMC5446171; doi:10.1371/journal.pone.0178380)
Supplement: S2 Table — The numbers correspond to p values of Fisher's exact tests with Bonferroni-Holm correction applied to the percentage of positive wells from each patient in comparison with non-infected subject, named MM, see Fig 1D and 1E. p<0.05 was considered significantly. (DOCX) [file pone.0178380.s005.docx]

**S2 Table:**

**Statistical analysis for the effect of PHA expansions on *T. cruzi* specific T cell response**

| **Challenge** | **Patient** | **IFN-γ** | **Proliferation** |
| --- | --- | --- | --- |
| 1st | RM25 | 1.000 | **<0.0001** |
|  | RM26 | **0.046** | **0.015** |
| 2nd | RM25 | 0.385 | 0.061 |
|  | RM26 | **0.001** | 0.473 |

The numbers correspond to *p* values of Fisher's exact tests with Bonferroni-Holm correction applied to the percentage of positive wells from each patient in comparison with non-infected subject MM, see Fig 1 D and E. *p*<0.05 was considered statistically significant.
