# Supplementary material for: Methodological approach to the ex vivo expansion and detection of T. cruzi-specific T cells from chronic Chagas disease patients
Source: PLoS One. 2017 May 26;12(5):e0178380. doi: 10.1371/journal.pone.0178380 (PMC5446171; doi:10.1371/journal.pone.0178380)
Supplement: S3 Table — The numbers correspond to p values of Fisher's exact tests with Bonferroni-Holm correction applied to the percentage of positive wells from the patient in comparison with non-infected subject, named MM, see Fig 3. p<0.05 was considered significantly. (DOCX) [file pone.0178380.s006.docx]

**S3 Table:**

**Statistical analysis for the effect of initial stimulus on memory CD4^+^ T cells**

| **Initial stimulus** | **Patient** | **IFN-γ** | **Proliferation** |
| --- | --- | --- | --- |
| *T. cruzi* | RM30 | **0.0007** | 0.153 |
| PHA | RM30 | **0.007** | 0.099 |

The numbers correspond to *p* values of Fisher's exact tests with Bonferroni-Holm correction applied to the percentage of positive wells from the patient in comparison with non-infected subject MM, see Fig 3. *p*<0.05 was considered statistically significant.
